# Strategic analysis of body composition indices and resting platelet ATP levels in professional soccer players for better platelet-rich plasma therapy

**DOI:** 10.3389/fbioe.2023.1255860

**Published:** 2023-08-30

**Authors:** Takashi Ushiki, Tomoharu Mochizuki, Katsuya Suzuki, Masami Kamimura, Hajime Ishiguro, Tatsuya Suwabe, Satoshi Watanabe, Go Omori, Noriaki Yamamoto, Tomoyuki Kawase

**Affiliations:** ^1^ Division of Hematology and Oncology, Graduate School of Health Sciences, Niigata University, Niigata, Japan; ^2^ Department of Transfusion Medicine, Cell Therapy and Regenerative Medicine, Niigata University Medical and Dental Hospital, Niigata, Japan; ^3^ Department of Hematology, Endocrinology and Metabolism, Faculty of Medicine, Niigata University, Niigata, Japan; ^4^ Department of Orthopaedic Surgery, Graduate School of Medical and Dental Sciences, Niigata University, Niigata, Japan; ^5^ Department of Orthopaedic Surgery, Niigata Medical Center, Niigata, Japan; ^6^ Department of Health and Sports, Faculty of Health Sciences, Niigata University of Health and Welfare, Niigata, Japan; ^7^ Department of Orthopaedic Surgery, Niigata Rehabilitation Hospital, Niigata, Japan; ^8^ Division of Oral Bioengineering, Graduate School of Medical and Dental Sciences, Niigata University, Niigata, Japan

**Keywords:** soccer player, sedentary, platelet, ATP, body composition, basal metabolic rate

## Abstract

**Background:** Autologous platelet-rich plasma (PRP) therapy is ambiguously thought to be more effective in elite athletes than in sedentary patients, although the possible importance of recipient responsiveness remains poorly understood. To address this issue, along with the well-known PRP quality, in this initial study, we evaluated two candidate biomarkers: body composition indices (BCIs), which reflect systemic physical conditions, and resting platelet ATP levels, which reflect platelet energy expenditure and the mass of energy generation units.

**Methods:** In this cross-sectional cohort study, blood samples were collected from male professional soccer players (PSPs) on a local professional team during the off-season and platelet ATP levels were quantified using an ATP luminescence assay kit. BCIs were measured using the body mass impedance method. Age-matched male sedentary participants were used as the controls.

**Results:** Among the BCIs, the body mass index, basal metabolic rate (BMR), and skeletal muscle weight levels were higher in the PSPs than in the controls. The platelet ATP levels in the PSPs group were significantly lower than those in the control group. The correlation between BMR and platelet ATP levels was moderately negative in the control group, but weakly positive in the PSPs group.

**Conclusion:** Owing to regular physical exercise, PSPs had higher BMR levels and lower platelet ATP levels without a significant mutual correlation compared to sedentary controls. This study did not indicate the influence of these biomarkers on the success of PRP therapy but provided evidence for a better understanding of PRP therapy, particularly for elite athletes.

## 1 Introduction

Autologous platelet-rich plasma (PRP) therapy is widely used in various regenerative therapies, for both medical and economic reasons. Although its medical basis is not yet well established (Kawase, 2015; Kawase et al., 2020), past clinical experience has brought a positive impression that influences patients’ and clinicians’ decisions. The major advantages of PRP therapy include minimal surgical invasion and injury. Furthermore, PRP therapy is ambiguously considered (or overestimated) more effective in elite athletes than in sedentary patients (CBC_NEWS, 2017). Therefore, professional elite athletes are increasingly choosing PRP therapy for the treatment of muscle or tendon injuries (Grambart, 2015; Mlynarek et al., 2016).

Regarding the medical basis for PRP’s effectiveness of PRP, it has been thought that growth factors, such as platelet-derived growth factor and vascular endothelial growth factor, are present at higher levels in platelets acting on cells at the site of injury (Kawase, 2015). However, clinicians have also experienced unsuccessful treatment regardless of platelet growth factor levels (Chalidis et al., 2023). This reminds us of two possibilities: one is the involvement of other known or unknown factors in PRP, which is generally evaluated as PRP quality, while the other is the recipient’s systemic or local condition. According to a recently proposed concept (Kawase, 2022), the regenerative potential of PRP therapy, which is a biomedical reaction utilized as adjuvant therapy, can be enhanced by previous or simultaneous treatments. In addition, systemic and local conditions could be basic factors that influence the success of PRP therapy. However, to date, this topic has been rarely studied or discussed.

**FIGURE 1:**
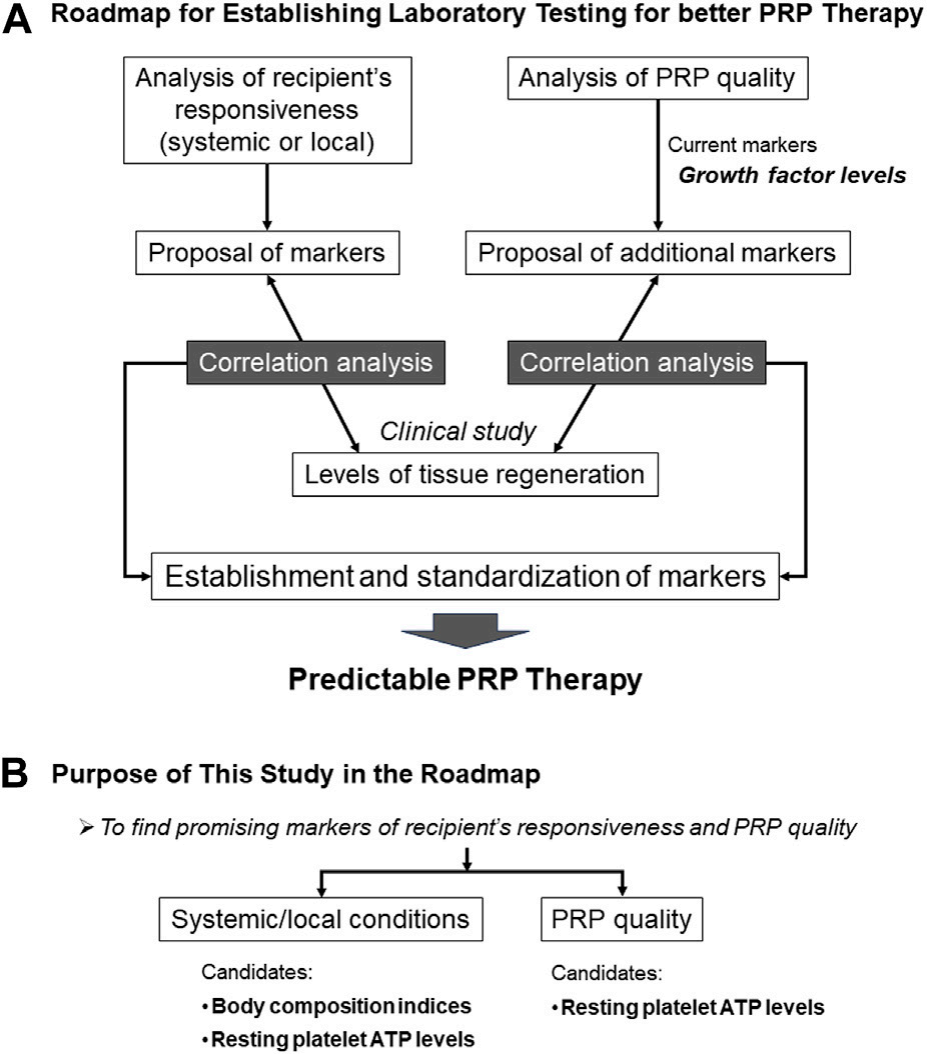
Grand design and ultimate goal of our PRP project **(A)** and the purpose of this study in the project **(B)**.

To redress this imbalanced understanding, we planned a roadmap to comprehensively understand how PRP works (Figure 1A). In the initial phases, candidate biomarkers for recipient responsiveness and PRP quality were listed and screened using appropriate preclinical experimental systems. The screened candidate biomarkers were further examined using correlation analysis with clinical indices in clinical studies. Subsequently, promising candidate biomarkers were validated in subsequent clinical studies.

This initial study was designed to screen for biomarkers of systemic recipient condition and PRP quality (Figure 1B). Systemic conditions can be evaluated based on the metabolic activity. Athletes do regular physical exercise, including muscle training, to improve their physical performance by increasing muscle mass and strength. Basal metabolic rate (BMR) usually correlates with body fat level (Yildirim et al., 2020); however, muscle mass also increases BMR (Zurlo et al., 1990). Thus, supplementally, in combination with other body composition indices (BCIs), BMR is expected to distinguish athletes from sedentary patients.

A recent study suggested that platelet bioenergetics correlate with muscle energetics and that skeletal muscle metabolic activity can be monitored by platelets without muscle tissue biopsy (Braganza et al., 2019). Resting platelet ATP levels can be considered a biomarker of local conditions. However, we focused on the recently proposed concept of intercellular mitochondrial transfer (Liu et al., 2021) and examined platelet ATP levels as biomarkers of PRP quality in this study.

The purpose of this initial preclinical study was to identify candidate biomarkers whose muscle levels differed substantially between the two groups. Accordingly, we tested BCIs and platelet ATP levels in the subsequent clinical studies.

## 2 Materials and methods

### 2.1 Participants and study design

A cross-sectional study was performed in two independent groups of healthy male adults (aged 19–37  years); the professional athlete group was composed of professional soccer players (PSPs) who were mainly Japanese and played in the domestic professional soccer league (J1 League), while an age-matched control group was composed of sedentary healthy adults who did not perform regular physical exercise. The inclusion criteria for the control group were as follows: healthy male young adults who were nonsmokers, had no systemic diseases regardless of medical control, underwent no daily physical training, and agreed to provide informed consent. Exclusion criteria included acute or chronic inflammatory conditions reflected in blood cell counts, and current or former thrombotic or platelet disorders. The inclusion and exclusion criteria for the PSP group were identical to those for the control group, with an additional criterion of continuous daily physical training.

Because this was not an interventional or observational clinical study, no participants in either the control or athlete group were treated with PRP.

The study design and consent forms for all procedures (approval no. 2021–0126) were approved by the Ethics Committee for Human Participants of Niigata University and complied with the Helsinki Declaration of 1964, as revised in 2013. The participants signed informed consent forms.

### 2.2 Blood collection and preparation of platelet suspensions

Blood was collected from participants between meals in glass vacuum tubes containing ACD-A (Vacutainer, Becton, Dickinson, and Company, Franklin Lakes, NJ, USA) using standard winged needle sets (21G) (Nipro, Osaka, Japan). Whole blood samples were transported from the hospitals to the laboratory by a parcel delivery service at ambient temperature (approximately 3°C–10°C). Before preparing the platelet suspensions, the samples were prewarmed for 2 h at 20°C–25°C to restore the platelets in the resting state. Platelet pellets were prepared using the double-spin method, gently suspended in phosphate-buffered saline (PBS) to prepare platelet suspensions, and stored at −80 °C until use, as described previously (Ushiki et al., 2022a; Ushiki et al., 2022b).

### 2.3 Blood cell counting

Blood cell counts were performed using an automated hematology analyzer (pocH iV-diff, Sysmex Corporation) before centrifugation to prepare the platelet suspension and before determining platelet ATP levels to adjust the platelet counts (Mochizuki et al., 2022). In addition to cell counting, data on histograms of platelet volume and mean platelet volume (MPV) were obtained. If the histograms of platelet distribution did not display a smooth curve, the samples were discarded and not subjected to subsequent experiments.

### 2.4 Determination of platelet ATP levels

The number of non-fixed living platelets suspended in PBS (100 μL) was adjusted to a density ranging between 40 and 60 × 10^4^/µL and stored at −80°C until use, usually within 2 weeks. After thawing, platelet ATP levels were determined using a luminescence ATP assay kit and luminescencer (AB-2200, Atto Corp., Tokyo, Japan).

### 2.5 Determination of body composition

Before blood collection, body composition of the participants was determined using a bathroom weighing scale (HCS-FS03; ECLEAR, ELECOM). This scale was installed with a unique MRI-based program that enables a more accurate evaluation of individual body fat percentage (BFP) based on measured bioelectrical impedance and body weight (So et al., 2012). Body mass index (BMI), BFP, and basal metabolic rate (BMR) were automatically determined using this weight scale. Body fat weight (BFW) and skeletal muscle weight (SMW) were calculated manually based on BFP, SMP, and body weight data.

Individual BMR values were calculated using the Mifflin-St Jeor equation, which is based on the number of calories burned while the body is at complete rest (Mifflin et al., 1990). For males, the formula shown below was used, and the scale factor for activity level was applied 1.2 and 1.725 for sedentary control and active PSPs, respectively (Medscape, 2020).

$$BMR=scale\;factor\times \left[\left(10\times weight\left[kg\right]\right)+\left(6.25\times height\left[cm\right]\right)–\left(5\times age\left[years\right]\right)+5\right]$$

### 2.6 Statistical analysis

To compare each index between the two groups, data were expressed as box plots, and the Mann–Whitney U test was performed to confirm statistical differences in the median and spread (SigmaPlot version 14.5; Systat Software, Inc., Systat Software, Inc.). Speaman’s rank correlation analysis was performed to compare the correlation between the two indices, and correlation coefficients were calculated using SigmaPlot software. Differences were considered statistically significant at *p* < 0.05.

The strength of the correlation was defined as very strong (0.8–1.0), strong (0.6–0.79), moderate (0.4–0.59), weak (0.2–0.39), and very weak (0–0.19).

## 3 Results

**FIGURE 2:**
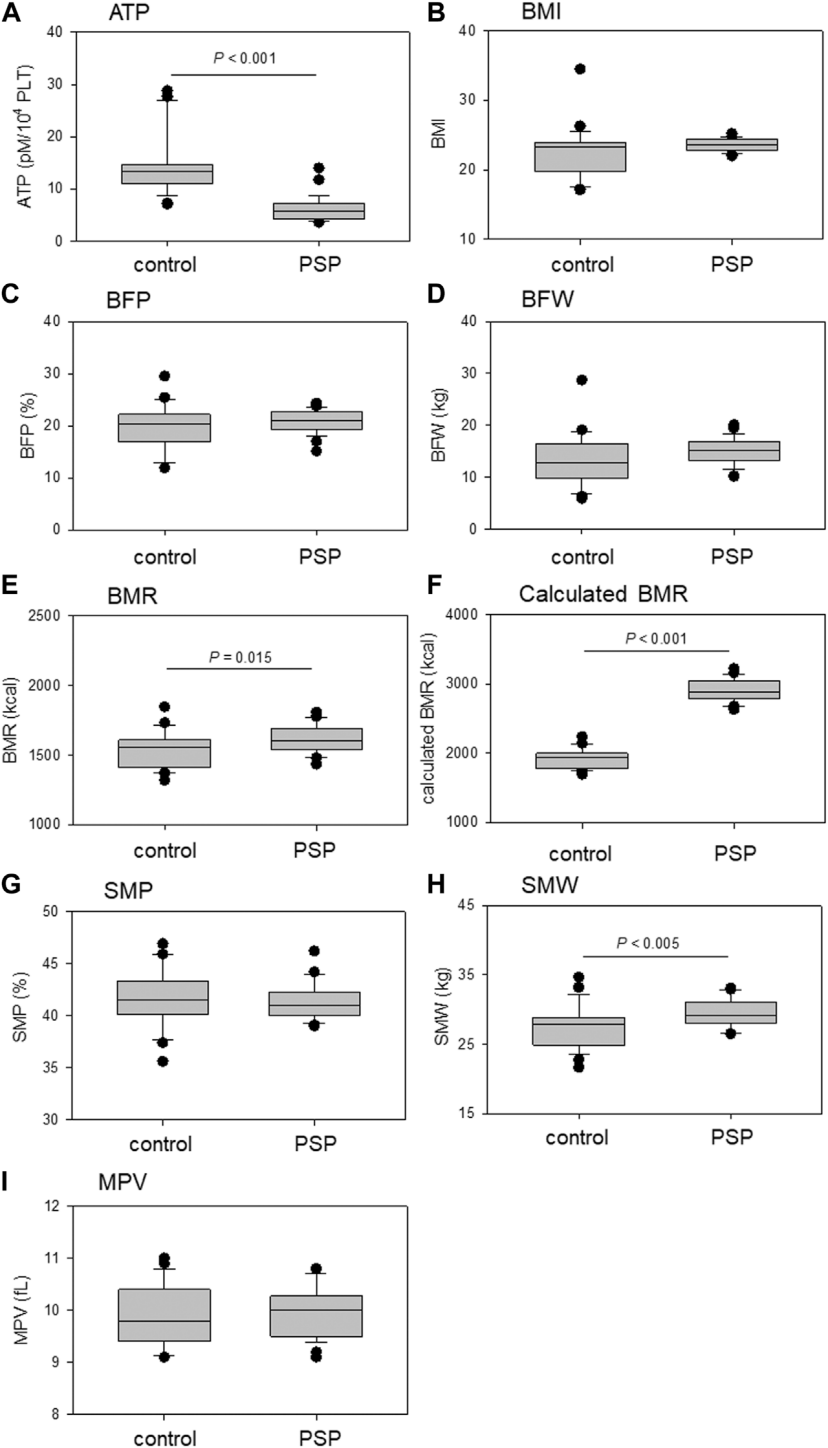
Comparison of platelet ATP **(A)**, mean platelet volume (MPV) **(I)**, and body composition indices (BCIs), including body mass index (BMI) **(B)**, body fat percentage (BFP) **(C)**, body fat weight (BFW) **(D)**, basal metabolic rate (BMR) **(E)**, calculated BMR **(F)**, skeletal muscle percentage (SMP) **(G)**, and skeletal muscle weight (SMW) **(H)** between sedentary control and professional soccer player (PSP) groups. *n* = 23 (control) or 28 (PSPs). Statistical analyses were performed using the non-parametric Mann–Whitney U test.

Comparisons of platelet ATP levels, MPV, and BCIs are shown in Figure 2. Platelet ATP levels were significantly lower in the PSP group than in the control group (*p* < 0.001). BMR, calculated BMR, and SMW were significantly higher in the PSP group than in the control group (*p* ≤ 0.015). In addition, the BMI of the PSP group tended to be higher than that of the control group (*p* = 0.057).

**FIGURE 3:**
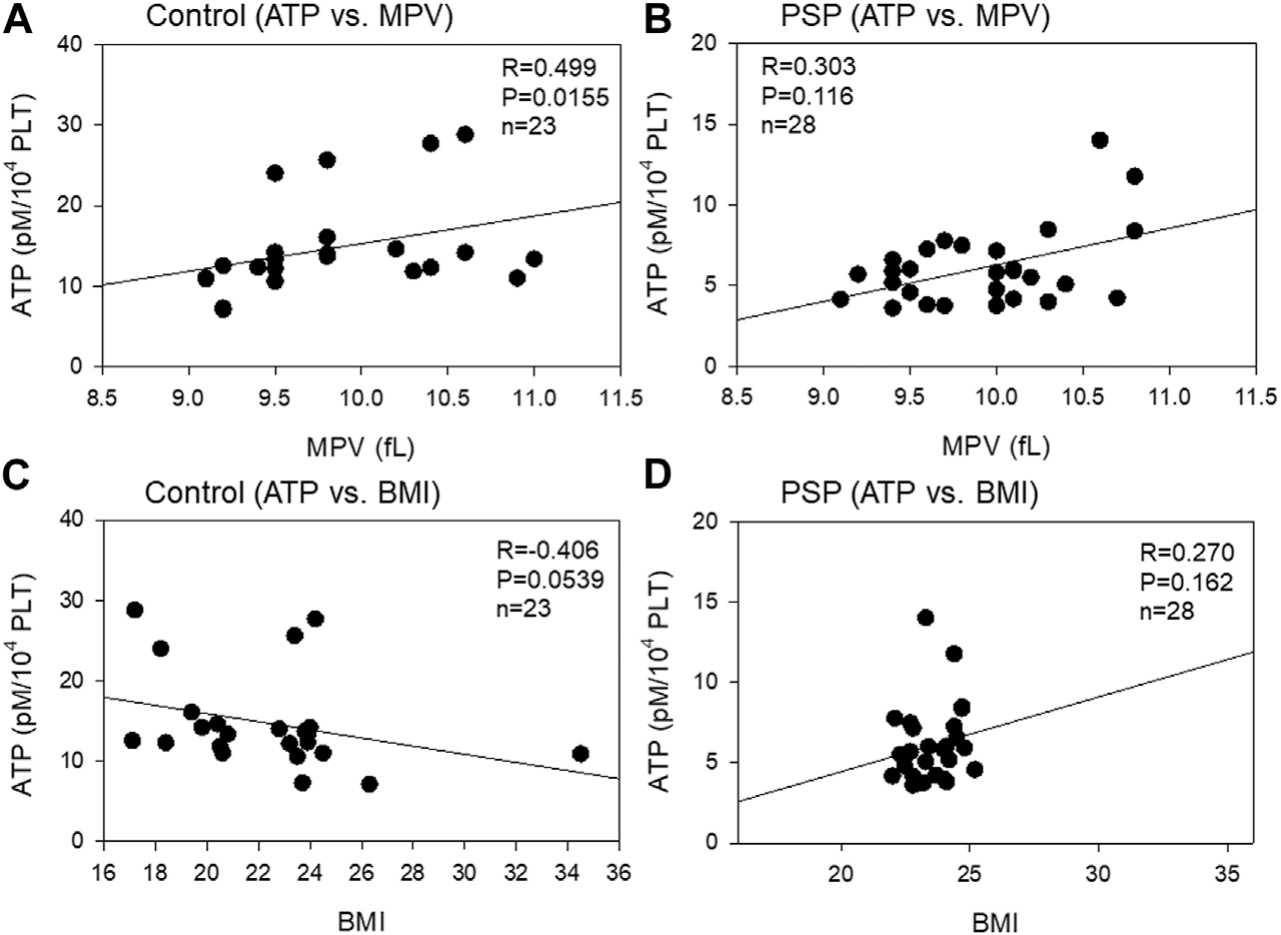
Correlations between platelet ATP levels and mean platelet volume (MPV) **(A, B)** or body mass index (BMI) **(C, D)** in the sedentary control **(A, C)** and professional soccer player (PSP) groups **(B, D)**. n = 23 (control) or 28 (PSPs). Spearman’s correlation coefficient (R) and probability (P) were used to evaluate the strength of the correlation.

The correlations between platelet ATP levels and MPV and BMI are shown in Figure 3. Moderate (R = 0.499, *p* = 0.0155) and weak (R = 0.303, *p* = 0.116) positive correlations were observed between ATP levels and MPV in the control and PSP groups, respectively. Overall, in the correlations between platelet ATP and BCIs, contradictory tendencies were observed in PSP compared with the control. A moderate (R = −0.406, *p* = 0.0539) negative correlation was observed between platelet ATP and BMI in the control group, while a weak positive correlation (R = 0.270, *p* = 0.162) was observed between these indices in the PSP group.

**FIGURE 4:**
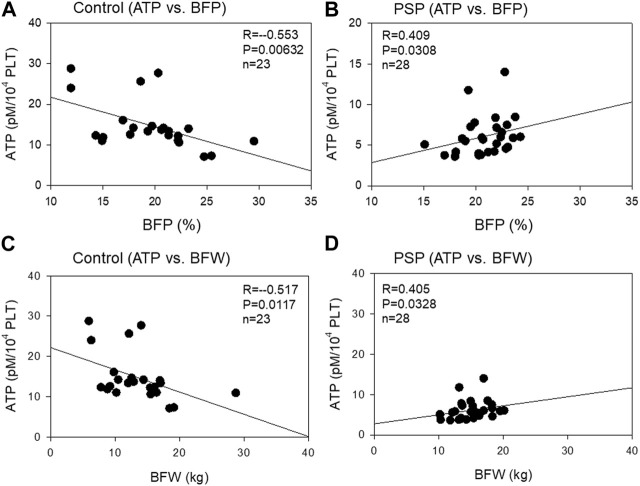
Correlations between platelet ATP and body fat percentage (BFP) **(A, B)** and body fat weight (BFW) **(C, D)** in the sedentary control **(A, C)** and professional soccer player (PSP) groups **(B, D)**. *n* = 23 (control) or 28 (PSPs). Spearman’s correlation coefficient (R) and probability (P) were calculated to evaluate the strength of the correlation.

The correlation between platelet ATP levels and body fat mass is shown in Figure 4. A moderate (R = −0.553, *p* = 0.00632) negative correlation was observed between platelet ATP and BFP in the control group, while a moderate (R = 0.409, *p* = 0.0308) positive correlation was observed between platelet ATP and BFP. Similar correlations were observed between platelet ATP and BFW in both the control (moderate: R = −0.517, *p* = 0.0117) and PSP (moderate: R = 0.405, *p* = 0.0328) groups.

**FIGURE 5:**
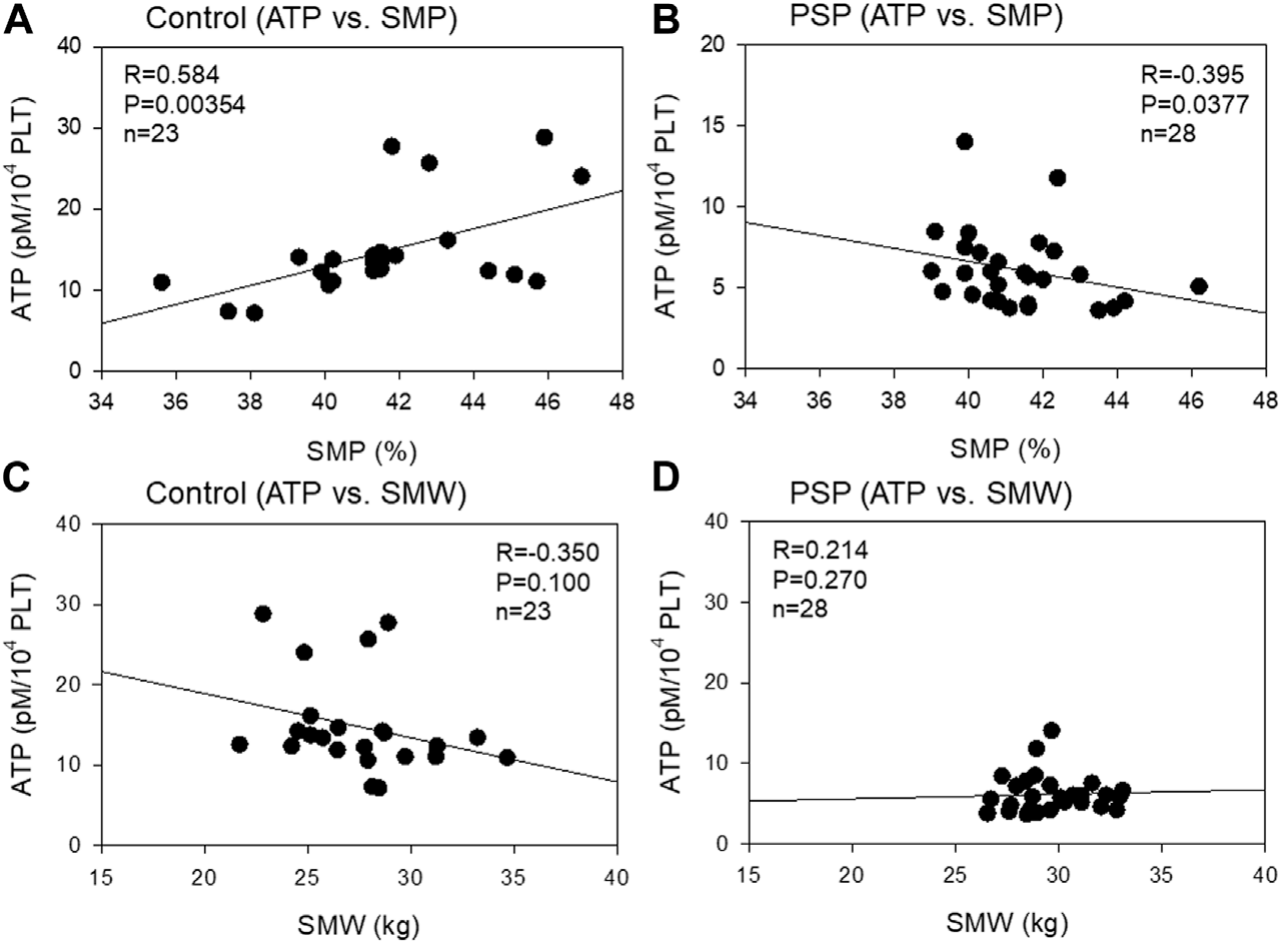
Correlations between platelet ATP and skeletal muscle percentage (SMP) **(A, B)** and skeletal muscle weight (SMW) **(C, D)** in sedentary control **(A, C)** and professional soccer player (PSP) groups **(B , D)**. *n* = 23 (control) or 28 (PSPs). Spearman’s correlation coefficient (R) and probability (P) were calculated to evaluate the strength of the correlation.

The correlation between platelet ATP levels and skeletal muscle mass is shown in Figure 5. A moderate (R = 0.584, *p* = 0.00354) negative correlation was observed between platelet ATP and SMP in the control group, while a weak (R = −0.395, *p* = 0.0377) positive correlation was observed between platelet ATP and SMP. In contrast, a weak (R = −0.350, *p* = 0.100) negative correlation was observed between platelet ATP and SMW in the control, while a weak (R = 0.214, *p* = 0.270) positive correlation was observed between these indices in PSP.

**FIGURE 6:**
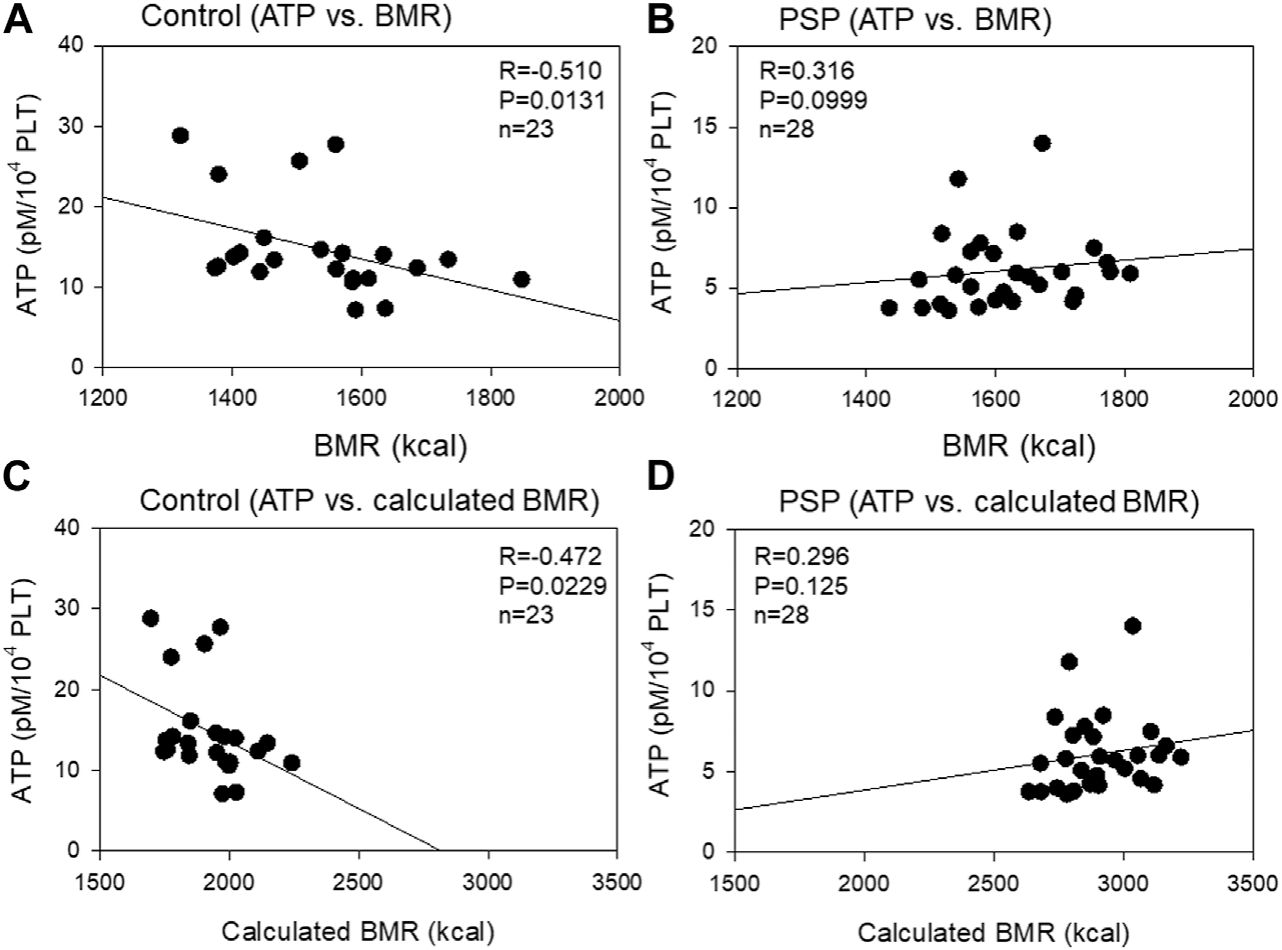
Correlations between platelet ATP levels and basal metabolic rate (BMR) **(A, B)** or calculated BMR **(C, D)** in the sedentary control **(A, C)** and professional soccer player (PSP) groups **(B, D)**. *n* = 23 (control) or 28 (PSPs). Spearman’s correlation coefficient (R) and probability (P) were calculated to evaluate the strength of the correlation.

The correlation between platelet ATP levels and BMR is shown in Figure 6. A moderate (R = −0.510, *p* = 0.0131) negative correlation was observed between platelet ATP and BMR, which does not consider individual physical activity, in the control group, while a weak (R = 0.316, *p* = 0.0999) positive correlation was observed between platelet ATP and calculated BMR, which is considered an individual physical activity. Similar correlations were observed between platelet ATP and calculated BMR in both control (moderate: R = −0.472, *p* = 0.0229) and PSP (weak: R = 0.296, *p* = 0.125).

## 4 Discussion

PRP therapy was developed to mimic the wound-healing process. The mechanism of PRP action underlying accelerated tissue regeneration has been explained solely by the condensed growth factor cocktail theory. However, if this theory can be applied to all cases, no one understands why there has been an endless dispute regarding the clinical effectiveness of PRP therapy. Thus, we hypothesized that PRP action could be modulated by known or unknown endogenous factors in PRP (i.e., the PRP quality) or by the recipient’s conditions (i.e., the recipient’s responsiveness) and planned to screen possible candidate biomarkers in a step-by-step manner to eventually reach the key factors.

BMR levels were higher in PSPs than in controls, and the correlations between platelet ATP levels and individual BCI levels, such as BMI, BFP, BFW, SMP, SMW, and BMR, in PSPs were distinguishable from those in controls. In contrast, resting platelet ATP levels were lower in PSPs than in sedentary controls. In previous studies (Ushiki et al., 2022b; Mochizuki et al., 2022), we observed that PDGF-BB, VEGF, and polyphosphate, which function as reservoirs of ATP in the cytoplasm (Müller et al., 2019), are stored at lower levels in platelets in the PSPs than in the controls. Superficially, the sum of these data does not seem to support a greater potential of PRP therapy in athletes.

### 4.1 Possible factors influencing BMR levels

BMR is the energy expenditure rate. BMR reflects the daily energy requirement for maintaining basic bodily functions (Ng and Schooling, 2023), which is a basic physical activity. Therefore, BMR is influenced by sex, race, exercise, diet, age, and various diseases (Nava and Raja, 2023). For example, some forms of physical activity, such as resistance training but not aerobic exercise, is reported to increase BMR levels (Ng and Schooling, 2023). Regardless of exercise, patients with type 2 diabetic mellitus generally have a higher BMR than healthy people (Ng and Schooling, 2023). In contrast, aging and progression of some other medical conditions may lower BMR levels over time (Ng and Schooling, 2023).

In this study, it could be judged easily by the other BCI levels and physical appearances that the increased BMR levels were due to the increased mass and/or strength of the skeletal muscle (Zurlo et al., 1990). In addition, this finding can be interpreted that such an organ possesses high power energy plants to meet the energy requirement and that its metabolic turnover is more active than that of low-BMR controls. To the best of our knowledge, no previous study has supported this hypothesis. However, because higher metabolic turnover could facilitate tissue regeneration, we chose BMR as a candidate biomarker for further investigation.

### 4.2 Possible factors influencing platelet ATP levels

Platelets are highly active cells and thus possess a highly potent unit of energy generation that is composed of mitochondrial oxidative phosphorylation and glycolysis (Kramer et al., 2014). In resting platelets, glycolysis is a major unit of ATP generation:65% of ATP is generated from glycolysis and 35% from oxidative phosphorylation (Ravi et al., 2015). Upon activation, glycolysis becomes more active in supplying energy (Aibibula et al., 2018; Kulkarni et al., 2019). High levels of generated ATP are required for morphological changes and aggregation upon activation and maintenance of intracellular ionic balance, particularly calcium ions (Petrus et al., 2019). In addition, phosphatase-dependent (Chaurasia et al., 2020) and -independent hydrolysis decreases platelet ATP levels regardless of the state.

The decrease in platelet ATP levels in resting PSPs suggests two possible mechanisms. First, the capacity of the ATP generation unit is suppressed. Second, ATP expenditure was maintained at relatively higher levels than that of ATP generation. From a physiological perspective, the first possibility seems less likely for PSPs. The second possibility is likely because the regular exercise the PSPs perform increases blood catecholamine levels to activate platelets (Messan et al., 2017), resulting in decreased platelet ATP levels. PSPs also frequently experience minor injuries that activate platelets for hemostasis and thrombogenesis during games and training.

We demonstrated in a previous study (Mochizuki et al., 2022) that, as were platelet ATP levels in this study, TGFβ1 and VEGF, growth factors stored in platelet α granules, were at lower levels in PSPs than in sedentary controls. As these growth factors are the major components involved in PRP-facilitated tissue regeneration, the decreased levels can be directly interpreted as the degradation of PRP quality. However, considering that these results are due to platelet activity and sensitivity, decreased levels of major platelet growth factors could indicate a higher intrinsic tissue regeneration potential.

Puzzlingly, a similar explanation can be made for the decreased platelet ATP levels. Recent studies have proposed a novel concept of mitochondrial transfer in tissue regeneration (Spees et al., 2016; Paliwal et al., 2018; Liu et al., 2021). This phenomenon is not found cell-specific and could occur between platelets and mesenchymal stem cells. According to the classic concept, when a tissue injury is detected, platelets gather to stop bleeding and release growth factors to facilitate tissue regeneration. In addition to these reactions, platelets are expected to transfer their mitochondria to cells, including mesenchymal stem cells, around the site of injury, to impel the cells for tissue regeneration. Thus, in addition to platelet growth factors, decreased platelet ATP levels may indicate higher intrinsic tissue regeneration potential. We leave platelet ATP level as an additional candidate biomarker for further investigation. The ultimate evaluation of platelet ATP and platelet growth factor levels would depend on clinical studies in the late phase of this project.

## 5 Conclusion

Owing to regular physical exercise, the PSPs had higher BMR levels and lower platelet ATP levels without a significant mutual correlation compared to sedentary controls. In the case of well-muscled athletes, because higher BMR levels reflect higher metabolic turnover, skeletal muscles may have a higher potential for tissue regeneration. Because lower platelet ATP levels reflect higher energy expenditure due to repeated activation, platelets may have a higher potential for the early detection and treatment of damaged tissues. These findings cannot directly suggest the validity of these biomarkers in predicting the success of PRP therapy but provide evidence for better understanding and improvement of PRP therapy.

## Data Availability

The raw data supporting the conclusion of this article will be made available by the authors, without undue reservation.
